# Metformin Suppresses Monocyte Immunometabolic Activation by SARS-CoV-2 Spike Protein Subunit 1

**DOI:** 10.3389/fimmu.2021.733921

**Published:** 2021-11-11

**Authors:** Theodore J. Cory, Russell S. Emmons, Johnathan R. Yarbro, Kierstin L. Davis, Brandt D. Pence

**Affiliations:** ^1^ Department of Clinical Pharmacy and Translational Science, College of Pharmacy, University of Tennessee Health Science Center, Memphis, TN, United States; ^2^ College of Health Sciences, University of Memphis, Memphis, TN, United States; ^3^ Department of Medicine, University of Tennessee Health Science Center, Memphis, TN, United States; ^4^ Center for Nutraceutical and Dietary Supplement Research, University of Memphis, Memphis, TN, United States

**Keywords:** COVID-19, SARS-CoV-2, immunometabolism, inflammation, monocyte

## Abstract

A hallmark of COVID-19 is a hyperinflammatory state associated with severity. Monocytes undergo metabolic reprogramming and produce inflammatory cytokines when stimulated with SARS-CoV-2. We hypothesized that binding by the viral spike protein mediates this effect, and that drugs which regulate immunometabolism could inhibit the inflammatory response. Monocytes stimulated with recombinant SARS-CoV-2 spike protein subunit 1 showed a dose-dependent increase in glycolytic metabolism associated with production of pro-inflammatory cytokines. This response was dependent on hypoxia-inducible factor-1α, as chetomin inhibited glycolysis and cytokine production. Inhibition of glycolytic metabolism by 2-deoxyglucose (2-DG) or glucose deprivation also inhibited the glycolytic response, and 2-DG strongly suppressed cytokine production. Glucose-deprived monocytes rescued cytokine production by upregulating oxidative phosphorylation, an effect which was not present in 2-DG-treated monocytes due to the known effect of 2-DG on suppressing mitochondrial metabolism. Finally, pre-treatment of monocytes with metformin strongly suppressed spike protein-mediated cytokine production and metabolic reprogramming. Likewise, metformin pre-treatment blocked cytokine induction by SARS-CoV-2 strain WA1/2020 in direct infection experiments. In summary, the SARS-CoV-2 spike protein induces a pro-inflammatory immunometabolic response in monocytes that can be suppressed by metformin, and metformin likewise suppresses inflammatory responses to live SARS-CoV-2. This has potential implications for the treatment of hyperinflammation during COVID-19.

## Introduction

The ongoing coronavirus disease 2019 (COVID-19) pandemic has presently claimed more than 3 million lives worldwide as of mid-April 2021 (1). COVID-19 is caused by a novel highly pathogenic coronavirus classified as severe acute respiratory syndrome coronavirus-2 (SARS-CoV-2) (2). A hallmark of severe COVID-19 is hyperinflammation (3), although cytokine expression patterns in individuals are diverse, leading to controversy over classification of COVID-19 related inflammation as cytokine storm, macrophage activation syndrome, multisystem inflammatory syndrome, etc. Regardless, inflammatory cytokines appear to play a principal role in mediating COVID-19 symptoms, therefore therapies which target these responses are paramount to treating severe COVID-19. As such, a fuller understanding of the cellular and molecular mechanisms mediating hypercytokinemia during SARS-CoV-2 infection is necessary.

Mononuclear phagocytes such as monocytes and macrophages are key constituents of the innate immune system, and produce pro-inflammatory cytokines during viral infection (4–7). We have previously suggested a key role for these cells in mediating severity of COVID-19 (7–9). Monocyte and monocyte-derived macrophage infiltration into the lungs has been linked to severe COVID-19 in single cell RNA sequencing studies (10–13) and postmortem analyses (14–17) in human patients, as well as during experimental infections in animal models including mice (18, 19), hamsters (20), and various non-human primates (21–25). Monocytes in individuals infected with SARS-CoV-2 display phenotypic changes associated with hyperinflammation, including reduced HLA-DR expression (26–28), increased CD16 expression (27, 29–31), and increased cytokine production (32–35). Both monocytes (36–38) and monocyte-derived macrophages (39, 40) also produce pro-inflammatory cytokines under direct infection with SARS-CoV-2, although infection at least in macrophages appears to be abortive (39, 40).

The past decade has seen an explosion in scientific interest in the regulation of immune cell activation and function by metabolic reprogramming. Under pro-inflammatory conditions, immune cells – including myeloid cells – generally undergo a switch to aerobic glycolysis which provides ATP sufficient to support cellular functions which propagate pro-inflammatory and anti-pathogen host responses (41). Recently, Codo et al. demonstrated pro-inflammatory glycolytic reprogramming in monocytes infected with SARS-CoV-2 (36), and SARS-CoV-2 also appears to alter monocyte lipid metabolism to promote lipid droplet formation which is associated with pro-inflammatory cytokine production (37).

SARS-CoV-2 therefore appears to reprogram metabolism in monocytes, but the viral factors which mediate these responses are unclear. Research in the 2003 epidemic SARS-CoV-1 suggested that the viral spike protein could mediate pro-inflammatory activation in macrophages (42, 43), and recent evidence suggests the spike protein of SARS-CoV-2 also activates inflammatory responses in macrophages and monocytes both *in vitro* and *in vivo* (44, 45). Given this, we hypothesized that spike protein binding to monocytes mediates glycolytic reprogramming to promote pro-inflammatory responses of these cells to SARS-CoV-2. Our results herein support this hypothesis, and we additionally report outcomes from experiments aimed at evaluating the responsible cellular signaling mechanisms, as well as potential pharmaceutical strategies for inhibiting these responses.

## Materials and Methods

### Subjects

Healthy 18–35-year-old subjects (N=14) were recruited without respect to sex or race. Participants reported to the laboratory approximately every two weeks for blood collection, and 8-24 ml blood was collected into EDTA-treated vacutainer tubes by venipuncture. Blood was immediately used for cell isolations as described below.

### Cell Isolations

Assays were performed on purified human classical monocytes isolated using immunomagnetic negative sorting (EasySep Direct Human Monocyte Isolation Kit, StemCell Technologies, Cambridge, MA). As we have previously described (46), this procedure results in a highly pure (> 85%) population of classical monocytes, with depletion of intermediate and non-classical monocytes due to the presence of an anti-CD16 antibody in the cocktail. Isolation purity was verified at several points throughout the current study and averaged approximately 90% (not shown). Cells were counted at 10× dilution using a Scepter cell counter (Millipore Sigma, St. Louis, MO). Isolated monocytes were immediately utilized in downstream assays, and no cells were frozen for later use.

### Media and Reagents

Unless otherwise specified, all assays were performed using Seahorse XF base DMEM medium (Agilent, Santa Clara, CA) supplemented with 10 mM glucose and 2 mM L-glutamine (Millipore Sigma, St. Louis, MO). Assays utilizing glucose deprivation omitted glucose from the media preparation. Media was not supplemented with fetal bovine serum or other additives. Recombinant spike protein subunit 1 (S1) was purchased from RayBiotech (Peachtree Corners, GA). 2-deoxyglucose, chetomin, compound C, and metformin were purchased from Millipore Sigma (St. Louis, MO). SARS-CoV-2 WA1/2020 strain was provided by Dr. Colleen Jonsson, Regional Biocontainment Laboratory, University of Tennessee Health Science Center.

### Seahorse Extracellular Flux

Glycolysis and oxidative phosphorylation were respectively quantified *via* kinetic monitoring of extracellular acidification rate (ECAR) and oxygen consumption rate (OCR) on a Seahorse XFp analyzer (Agilent, Santa Clara, CA). For all assays, monocytes were plated at 1.5×10^5^ cells per well, and wells A and H of the XFp plate were background wells with no cells. All analyses were run in duplicate. Plated cells were incubated at 37°C in a non-CO_2_ incubator for 1 hour prior to assays to stabilize pH. All wells were imaged at 10× magnification for cell counting in order to adjust raw measurements for cell number.

For quantification of dose response to S1, 5 basal measurements were made, followed by injection of media (wells B-C), 100 nM spike protein (wells D-E), or 300 nM spike protein (wells F-G). After injection into existing media in the well, spike protein concentrations were 10-fold lower than injection concentrations, thereby giving final spike protein concentrations of 0 nM, 10 nM, or 30 nM. Following injection, ECAR and OCR were monitored serially for 60 measurements. Following the assay, cell culture supernatants were removed, pooled by duplicate, and stored at -80°C. Cells were then lysed with 100 μl Trizol (Thermo Fisher Scientific, Waltham, MA), pooled by duplicate, and stored at -80°C as we have previously described (47).

For chetomin and metformin Seahorse assays, cells were incubated in media as above (wells B-E), or either 10 nM chetomin or 50 mM metformin during the 1-hour pre-incubation period (wells F-G). 5 basal ECAR/OCR measurements were performed, followed by injection of media (wells B-C) or 300 nM spike protein (wells D-G) for a final concentration of 0 nM (wells B-C) or 30 nM spike protein (wells D-G) as above. Following injection, ECAR and OCR were monitored serially for 30 measurements. Cell culture supernatants and Trizol lysates were processed as described above following the end of the assay.

For glycolysis inhibition assays, cells were incubated in media, 10 mM 2-deoxyglucose, or media without glucose (glucose deprivation) during the 1-hour pre-incubation period. 5 basal ECAR/OCR measurements were performed, followed by injection of 300 nM spike protein to all wells for a final concentration of 30 nM spike protein per well as above. Spike protein was prepared in non-glucose media for the glucose deprivation condition. Following injection, ECAR and OCR were monitored serially for 30 measurements. Cell culture supernatants and Trizol lysates were processed as described above following the end of the assay.

### SARS-CoV-2 Infections

Isolated monocytes were incubated in RPMI-1640 media (Gibco, Thermo Fisher Scientific, Waltham, MA) supplemented with 10% fetal bovine serum (Gibco), with or without 50 mM metformin, for 1-hour. Cells were then treated with media or infected with SARS-CoV-2 virus (WA1/2020 isolate) at 0.5 MOI and incubated for 24 hours. Cell culture supernatants were collected from untreated and infected cells and stored at -80°C until analysis. SARS-CoV-2 experiments were conducted under biosafety level 3 at the Regional Biocontainment Laboratory at the University of Tennessee Health Science Center.

### Gene and Protein Expression Analysis

RNA isolation was performed using the Trizol procedure based on manufacturer’s instructions from cells lysed directly in the microplate or Seahorse plate wells as applicable. Isolated RNA (300-400 ng depending on experiment) was reverse-transcribed to cDNA using a High-Capacity cDNA Reverse Transcription Kit (Thermo Fisher Scientific, Waltham, MA). Gene expression was analyzed using commercial pre-validated gene expression assays and Taqman reagents (Thermo Fisher Scientific, Waltham, MA). Relative gene expression was quantified using the 2^-ΔΔCt^ method (48) against *B2M* or *ACTB* as housekeeping genes. Primer/probe IDs were: *B2M* Hs00187842_m1; *ACTB* Hs03023943_g1; *IL1B* Hs01555410_m1; *IL6* Hs00174131_m1; *CXCL8* Hs00174103_m1; *TNF* Hs00174128_m1; *CPT1A* Hs00912671_m1; *G6PD* Hs00166169_m1; *HLA-DRA* Hs00219575_m1; *CDKN2A* Hs00923894_m1; *TLR4* Hs00152939_m1; *IL10* Hs00961622_m1; *TGFB1* Hs00998133_m1.

For protein quantification, cell culture supernatants harvested from microplates or Seahorse XFp plates were analyzed *via* ELISA. Commercial DuoSet matched-antibody reagent sets were purchased from R&D Systems (Minneapolis, MN) for quantifying human IL-6, human IL-1β, human IL-8, and human TNFα and were used according to manufacturer’s instructions. All samples were run in duplicate at 5× dilution (SARS-CoV-2 assays), or 50× dilution (Seahorse S1 dose response assays), or 10× dilution (all others) and assessed against a standard curve.

Protein concentration of angiotensin converting enzyme 2 (ACE2) and C-reactive protein (CRP) was performed by ELISA on plasma samples collected by venipuncture from subjects at the beginning of the study. Peripheral blood was collected by venipuncture into EDTA-coated vacutainer tubes, centrifuged at 1,500×*g* for 15 min, aliquoted, and stored at -80°C until analysis. Plasma samples were analyzed in duplicate at 10× (ACE2) or 10,000× (CRP) using commercial DuoSet matched-antibody reagent kits (R&D Systems) according to manufacturer’s instructions and assessed against a standard curve. ACE2 and CRP data are included in the FigShare repository (49), along with other clinical and anthropomorphic data for experimental subjects.

### Data Processing and Statistical Analysis

All data processing and statistical analyses were performed using R v. 3.6.2 (50). Isolated monocytes from each subject were given all treatments for each experiment, so data were paired and analyzed using within-subjects designs. Data were checked for normality by Shapiro-Wilk test and analyzed by one-way repeated measures ANOVA (RM-ANOVA, for data which met the normality assumption) or Friedman’s test (for data which did not meet the normality assumption). For analyses with significant main effects, *post hoc* mean separation was performed using pairwise paired T tests (for RM-ANOVA) or pairwise Wilcoxon signed-rank tests (for Friedman’s tests) with p-value adjustment using the Holm-Bonferroni method (51). Significance cutoff was p<0.05.

All raw data and analytical scripts (as R markdown files) are available in a dedicated FigShare repository (49). This manuscript was posted to the preprint server *bioRxiv* prior to submission for publication (52).

### Study Approval

All human subjects activities were approved by the Institutional Review Board at the University of Memphis under protocol 4316, and subjects provided written informed consent prior to enrollment.

## Results

### Spike Protein Subunit 1 Reprograms Metabolism and Promotes Inflammatory Responses

**Figure 1:**
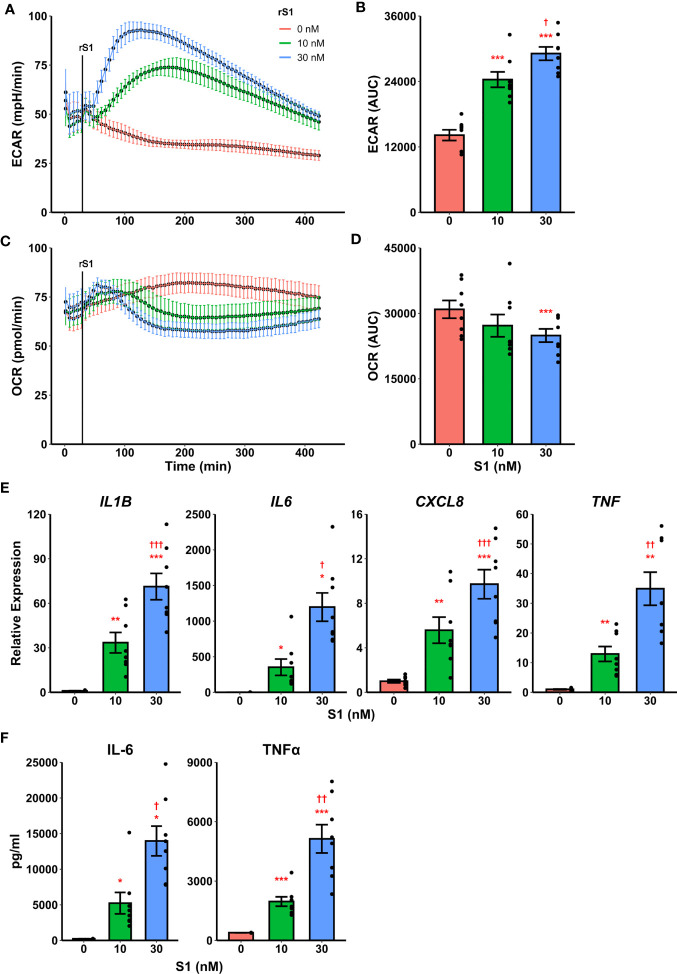
Recombinant SARS-CoV-2 spike protein subunit 1 (rS1) mediates immunometabolic activation of monocytes. **(A)** Monocytes increase extracellular acidification response rate (ECAR) in a dose-dependent manner when treated with rS1. **(B)** Quantification of ECAR by area under the curve (AUC). **(C)** rS1 treatment suppresses oxygen consumption rate (OCR) in monocytes in a dose-dependent fashion. **(D)** Quantification of OCR by AUC. **(E)** Gene expression analysis by qPCR reveals dose-dependent increases in responses of *IL1B*, *IL6*, *CXCL8*, and *TNF* to rS1 stimulation. **(F)** Protein expression analysis by ELISA reveals dose-dependent increases in responses of IL-6 and TNFα to rS1 stimulation. ECAR and OCR data in panels A-D are adjusted for values indexed to 1×10^5^ cells/well. *, **, ***p < 0.05, p < 0.01, p < 0.001 *vs*. 0 nM rS1. †, ††, †††: p < 0.05, p < 0.01, p < 0.001 *vs*. 10 nM rS1. N = 8 biological replicates. Bars and error bars are mean ± SEM.

Recently it was demonstrated that SARS-CoV-2 promotes metabolic reprogramming in monocytes during infection (36, 37). Research in SARS-CoV-1 suggested that the viral spike protein induces inflammatory responses in macrophages (42, 43), and this has recently been replicated using spike protein from SARS-CoV-2 (44, 45). Likewise, spike protein binding to C-type lectins has recently been shown to mediate pro-inflammatory processes in myeloid cells (53, 54). Therefore, we hypothesized that the SARS-CoV-2 spike protein mediates a pro-inflammatory metabolic reprogramming in monocytes which could be a basis for hypercytokinemia. Stimulation of isolated human classical monocytes with recombinant spike protein subunit 1 (S1) from SARS-CoV-2 induced glycolytic activation (**Figure 1A**) and suppressed oxidative phosphorylation (OXPHOS, **Figure 1C**) in a dose-dependent manner. The effect of S1 dose was significant for both extracellular acidification rate (F_2,14_ = 72.44, p<0.0001, **Figure 1B**) and oxygen consumption rate (F_2,14_ = 5.785, p=0.0147, **Figure 1D**) as measured by quantification of area under the response curve.

Additionally, recombinant S1 treatment caused a dose-dependent increase transcription of pro-inflammatory cytokines (**Figure 1E**) including *IL1B* (F_2,14_ = 50.98, p<0.001), *IL6* (Friedman χ^2^_(df=2)_=16, p<0.001), *CXCL8* (F_2,14_ = 38.19, p<0.001), and *TNF* (F_2,14_ = 28.41, p<0.001) as measured by qPCR. These cytokines have been implicated in the pathogenesis of SARS-CoV-2 and in COVID-19-related hypercytokinemia in several studies (27, 55–60). To confirm that increased transcription resulted in increased protein expression, we evaluated protein concentrations of key cytokines in the supernatant of S1-stimulated monocytes by enzyme-linked immunosorbent assay (ELISA) (**Figure 1F**). S1 increased protein expression of interleukin (IL)-6 (Friedman χ^2^_(df=2)_=16, p<0.001) and tumor necrosis factor (TNF)-α (F_2,14_ = 37.73, p<0.001) in a dose-dependent manner.

**Figure 2:**
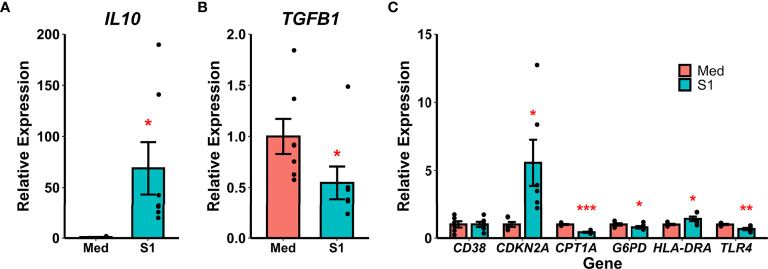
Recombinant SARS-CoV-2 spike protein subunit 1 (S1) alters expression of anti-inflammatory cytokine and polarization-related genes. **(A)** S1 treatment increased expression of *IL10*. **(B)** S1 treatment decreased expression of *TGFB1*. **(C)** S1 increased expression of *CDKN2A* and *HLA-DRA*, while decreasing expression of *CPT1A*, *G6PD*, and *TLR4*. *, *, ***p < 0.05, p < 0.01, p < 0.001 *vs*. media-treated cells. N = 7 biological replicates. Bars and error bars are mean ± SEM.

Recombinant S1 treatment also increased gene expression of the anti-inflammatory cytokine IL-10 (Wilcoxon *Z*=0, p=0.016, **Figure 2A**) and decreased gene expression of the anti-inflammatory cytokine TGF-β (Wilcoxon *Z*=28, p=0.016, **Figure 2B**). We additionally analyzed gene expression for a variety of polarization and reprogramming markers (**Figure 2C**), and found that rS1 treatment increased expression of the senescence-associated gene for p16 (*CDKN2A*, t_(df=5)_=-2.985, p=0.031) as well as *HLA-DRA* (t_(df=5)_=-2.887, p=0.034). Treatment with 30 nM rS1 also reduced expression of metabolism-related genes *G6PD* (Wilcoxon *Z*=21, p=0.031) and *CPT1A* (t_(df=5)_=15.373, p<0.001) and the LPS receptor *TLR4* (t_(df=5)_=4.833, p=0.005). Therefore, monocytes appear to be activated by S1 and upregulate expression of both pro- and anti-inflammatory cytokines, and additionally show gene expression patterns reflecting altered metabolism.

### Glycolytic Response to Spike Protein Is Dependent on HIF-1α

**Figure 3:**
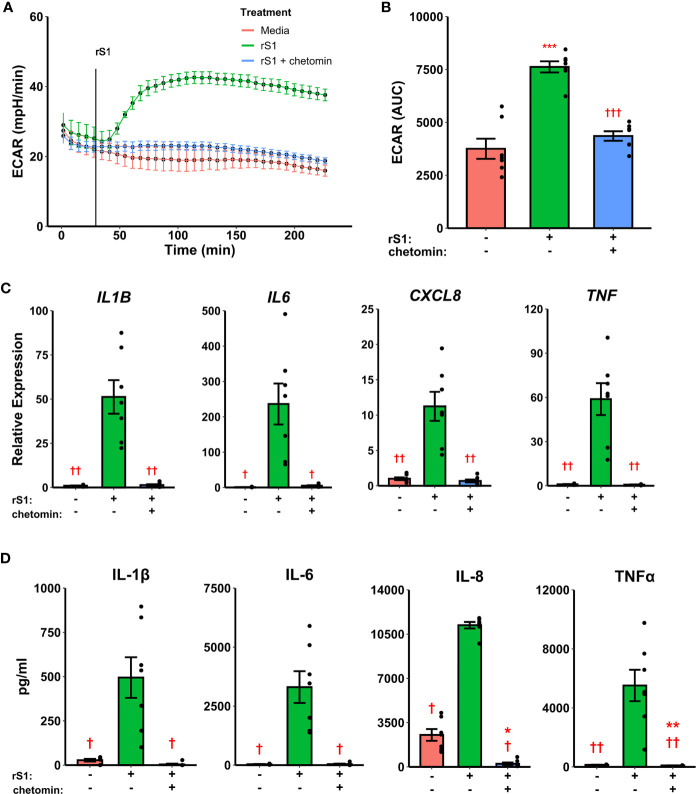
HIF-1α inhibition suppresses immunometabolic activation of monocytes due to recombinant spike protein (rS1). **(A)** Monocytes increase extracellular acidification response rate (ECAR) when treated with 30 nM rS1, but this is blocked by pre-treatment with chetomin. **(B)** Quantification of ECAR by area under the curve (AUC). **(C)** rS1 increase of expression of *IL1B*, *IL6*, *CXCL8*, and *TNF* is reversed by chetomin pre-treatment. **(D)** rS1 increase of IL-1β, IL-6, IL-8, and TNFα is reversed by chetomin pre-treatment. *, **, ***: p < 0.05, p < 0.01, p < 0.001 *vs*. untreated cells. †, ††, †††: p < 0.05, p < 0.01, p < 0.001 *vs*. rS1-treated cells. N = 7 biological replicates. Bars and error bars are mean ± SEM.

Hypoxia inducible factor (HIF)-1α was demonstrated nearly 20 years ago to mediate pro-inflammatory responses in myeloid cells (61), and has more recently been shown to regulate glycolytic activation in monocytes, macrophages, and other immune cells (62–64). SARS-CoV-2 activates HIF-1α-mediated glycolysis in monocytes (36), so we reasoned that this was a likely downstream mechanism by which the viral spike protein causes this similar glycolytic reprogramming in our experiments. As above, treatment of monocytes with S1 activated glycolysis, and this effect was abrogated by pre-treatment with chetomin (**Figures 3A, B**, F_2,12_ = 42.43, p<0.001), which disrupts the interaction between HIF-1α and p300 to block the effects of the former (65). Pre-treatment with chetomin also strongly suppressed the cytokine response due to S1 treatment (**Figure 3C**), including blunting transcription of *IL1B* (F_2,12_ = 27.35, p<0.001), *IL6* (F_2,12_ = 16.11, p<0.001), *CXCL8* (F_2,12_ = 25.54, p<0.001), and *TNF* (F_2,12_ = 29.04, p<0.001). Protein concentrations of these cytokines in culture supernatants showed similar patterns (**Figure 3D**). As such, HIF-1α appears to be a master regulator of both glycolytic reprogramming and inflammatory activation of monocytes under S1 stimulation.

### Suppression of Glycolysis Alters Inflammatory Responses to Spike Protein

**Figure 4:**
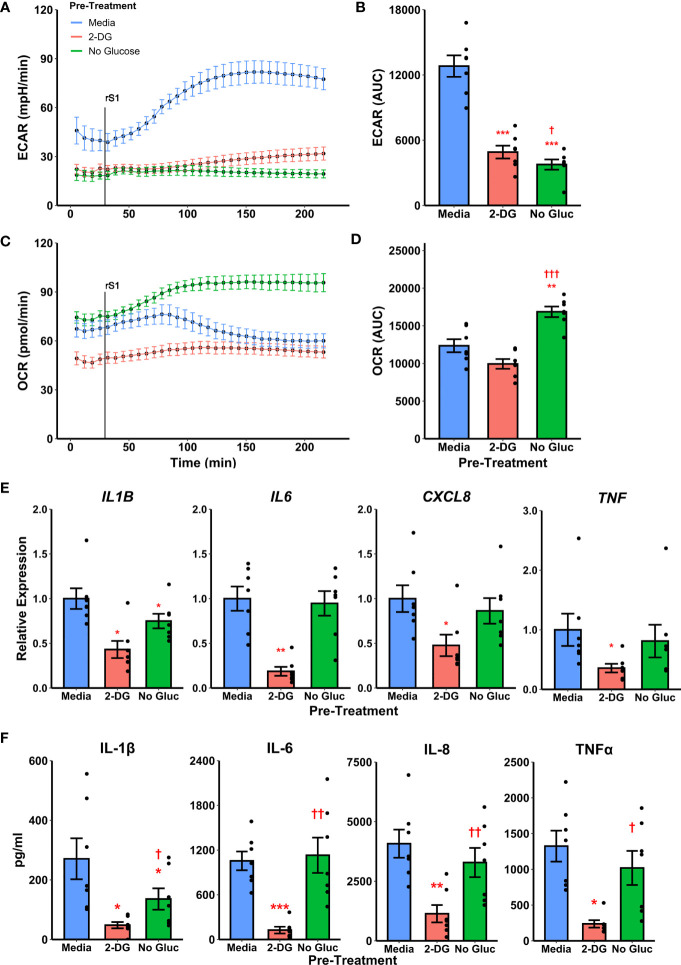
Targeting glycolysis has variable effects on recombinant spike protein (rS1) responses in monocytes. **(A)** Glucose deprivation or pre-treatment with 2-deoxyglucose (2-DG) block extracellular acidification rate (ECAR) increase due to rS1 treatment. **(B)** Quantification of ECAR by area under the curve (AUC). **(C)** 2-DG inhibits oxygen consumption rate in rS1-treated monocytes, but glucose-deprived monocytes upregulate OCR in response to rS1. **(D)** Quantification of OCR by AUC. **(E)** 2-DG blocks expression of *IL1B*, *IL6*, *CXCL8*, and *TNF* due to rS1 stimulation, but glucose deprivation has limited effects on cytokine expression. **(F)** 2-DG blocks protein production of pro-inflammatory cytokines, but glucose deprivation has limited effect. *, **, ***: p < 0.05, p < 0.01, p < 0.001 *vs*. rS1-treated cells. †, ††, †††: p < 0.05, p < 0.01, p < 0.001 *vs*. 2-DG-treated cells. N = 7 biological replicates. Bars and error bars are mean ± SEM.

To determine whether metabolic reprogramming is responsible for altered cytokine responses to S1, we suppressed glycolytic responses during S1 treatment using 2-deoxyglucose (2-DG) pretreatment. Treatment of monocytes with 2-DG ablated monocyte glycolytic responses to S1 stimulation (**Figure 4A**) which was significant by comparison of area under the response curve (t_6_=-10.867, p<0.0001, **Figure 4B**). However, 2-DG also suppressed mitochondrial function in these cells (**Figure 4C**), though this was non-significant by area under the oxygen consumption (t_6_=-2.2284, p=0.0674, **Figure 4D**). This effect has been noted previously during responses to LPS (66). Anticipating this, we also included a condition where monocytes were cultured under glucose deprivation, as a second method of suppressing glycolytic activation. We noted a similar ablation of glycolytic responses to S1 using this strategy (**Figure 4A**) which was significant by area under the curve analysis (t_6_=-14.045, p<0.0001, **Figure 4B**). However, glucose deprivation caused an increase in oxygen consumption after S1 treatment (**Figure 4C**) which was significant compared to media- (t_6_ = 4.6618, p=0.0069) or 2-DG (t_6_=-15.607, p<0.001) pretreated monocytes (**Figure 4D**).

Pre-treatment of monocytes with 2-DG also strongly inhibited cytokine expression compared to cells treated with S1 (**Figure 4E**), including transcription of *IL1B* (*W*=0, p=0.0313), *IL6* (t_6_=-5.912, p=0.0021), *CXCL8* (*W*=0, p=0.0313), and *TNF* (*W*=0, p=0.0313). However, glucose deprived monocytes generally maintained their ability to transcribe pro-inflammatory cytokines in response to S1, with only *IL1B* expression showing a modest 25.1% reduction in glucose deprived compared to S1-treated monocytes (**Figure 4E**, *W*=0, p=0.0313). Protein concentrations in cell culture supernatants followed gene expression patterns (**Figure 4F**). Monocytes appear to utilize fatty acid oxidation to compensate for loss of glycolysis during cytokine responses as has been previously demonstrated with LPS (66–69), and therefore the 2-DG-mediated suppression of S1-induced inflammation is likely due to its ability to suppress both glycolysis and mitochondrial metabolism in concert.

### Metformin Abrogates Inflammatory Response to Spike Protein

The small molecule compounds chetomin and 2-deoxyglucose inhibited immunometabolic activation in monocytes, suggesting a potential strategy for treating hypercytokinemia during COVID-19. However, chetomin is not approved for use in humans, although it has shown efficacy *in vivo* in animal models (65). Additionally, 2-DG has poor efficacy in humans due to rapid metabolism and limited bioavailability (70). Therefore, we investigated the ability of the common diabetes and geroprotector drug metformin to inhibit cytokine production in S1-stimulated monocytes. Metformin activates AMPK (71) and (independently of AMPK) opposes the action of HIF-1α (72, 73), and additionally inhibits mitochondrial metabolism through blocking complex I of the electron transport chain (74, 75), thus we hypothesized that it would have a qualitatively similar effect to 2-DG in inhibiting cytokine production through dual inhibition of glycolysis and OXPHOS.

**Figure 5:**
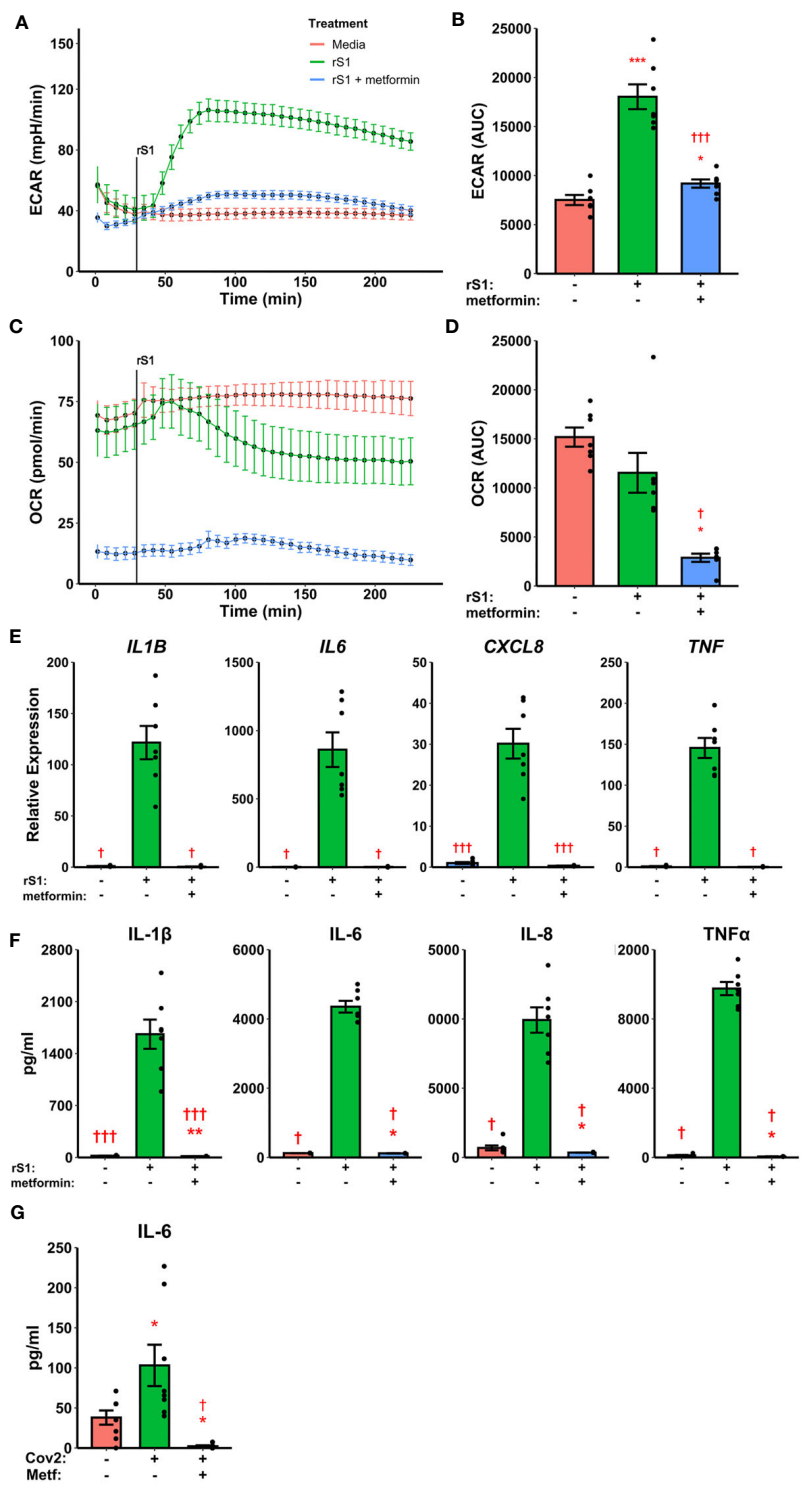
Metformin suppresses immunometabolic activation in monocytes treated with recombinant spike protein (rS1). **(A)** Metformin pre-treatment blocks the increase in extracellular acidification rate (ECAR) mediated by rS1. **(B)** Quantification of ECAR by area under the curve (AUC). **(C)** Metformin suppresses oxygen consumption rate (OCR). **(D)** Quantification of OCR by AUC. **(E)** Metformin suppresses cytokine responses, as demonstrated by gene expression of *IL1B*, *IL6*, *CXCL8*, and *TNF*, during rS1 stimulation in monocytes. **(F)** Metformin suppresses protein production of pro-inflammatory cytokines IL-1β, IL-6, IL-8, and TNFα during S1 stimulation in monocytes. **(G)** Metformin inhibits IL-6 production in monocytes infected with SARS-CoV-2 strain WA1/2020 (Cov2) at 0.5 MOI. *, **, ***: p < 0.05, p < 0.01, p < 0.001 *vs*. unstimulated cells. †, †††: p < 0.05, p < 0.001 *vs*. rS1-treated or Cov2-infected cells. N = 7-8 biological replicates. Bars and error bars are mean ± SEM.

Pre-treatment with metformin abrogated the glycolytic response to S1 in monocytes (**Figures 5A, B**, F_2,12_ = 60.05, p<0.001) and strongly inhibited cellular respiration (**Figures 5C, D**, Friedman χ^2^_(df=2)_=12.286, p=0.0021) in Seahorse assays. Likewise, metformin pre-treatment suppressed cytokine responses to S1 treatment in monocytes (**Figure 5E**), including *IL1B* (Friedman χ^2^_(df=2)_=12.286, p=0.0021), *IL6* (Friedman χ^2^_(df=2)_=10.571, p=0.0051), *CXCL8* (F_2,12_ = 68.18, p<0.0001), and *TNF* (Friedman χ^2^_(df=2)_=12.286, p=0.0021). As in previous experiments, protein concentrations (**Figure 5F**) followed gene expression patterns.

### Metformin Abrogates IL-6 Production in Virus-Stimulated Monocytes

Recent evidence suggests that myeloid cells recognize SARS-CoV-2 spike protein through C-type lectins (53, 54). However, the SARS-CoV-2 virion also contains additional immunoregulatory and pro-inflammatory proteins (76, 77), therefore we examined the ability of metformin to block cytokine responses to live SARS-CoV-2. Monocytes treated for 24 hr with SARS-CoV-2 increased expression of IL-6 protein, and this was suppressed by metformin pre-treatment (**Figure 5G**, F_2,14_ = 11.48, p=0.0011), suggesting that the anti-inflammatory effect of metformin is generalizable to SARS-CoV-2 infection of monocytes.

## Discussion

The present study resulted in several advances of major importance for the understanding of SARS-CoV-2 innate immune responses. First, we report here that monocytes treated with recombinant spike protein subunit 1 from the current pandemic SARS-CoV-2 undergo a dose-dependent increase in glycolysis which can be suppressed by a HIF-1α inhibitor and mediates the production of pro-inflammatory cytokines. These data suggest an initial signaling event which precipitates changes in glucose and lipid metabolism during SARS-CoV-2 infection in monocytes which have been previously reported to be linked to inflammatory activation (36, 37). Monocyte and monocyte-derived macrophages are substantially enriched in the lungs of SARS-CoV-2-infected individuals with severe COVID-19 (10, 11, 14–17) and respond to experimental viral infection by producing pro-inflammatory cytokines (36–40), therefore these results reflect a potential mechanism by which hypercytokinemia occurs during the early innate immune response to SARS-CoV-2.

Importantly, the available evidence suggests that infection of monocytes/macrophages by SARS-CoV-2 is abortive (39, 40, 78), thus recognition of SARS-CoV-2 structural proteins or genomic material is the likely mechanism by which direct infection precipitates inflammatory responses in this cell type. Our data suggest the spike protein is one such determinant, although we cannot conclude it is the only such mechanism given that recent reports have demonstrated inflammatory responses in macrophages treated with the SARS-CoV-2 envelope protein (76). It is also possible, however, that direct viral binding to monocytes is not the only way in which these cells can be exposed to the viral spike protein. Recent evidence suggests that vaccine antigens including S1 are released into the circulation following vaccination (albeit at very low levels) (79), and this represents a potential pro-inflammatory stimulus for monocytes. Monocyte/macrophage recognition of S1 may also contribute to the local (muscle) inflammatory response during vaccination. Additionally, the viral spike protein undergoes cleavage by furin during binding to ACE2 (80), and it has been suggested that this could lead to release of the S1 subunit during infection (81), although to date this is speculative.

ACE2 has limited expression on immune cells including monocytes and macrophages (82), which has called into question whether they can directly recognize SARS-CoV-2. In this study we did not identify the mechanism for monocyte recognition of S1, but several recent papers have shed light on this. Two reports recently demonstrated spike binding to C-type lectin receptors (53, 54) which mediates pro-inflammatory signaling in myeloid cells. Likewise, monocytes and macrophages express high levels of CD147 (82), and this receptor has been shown to recognize spike protein and contribute to activation of T cells (83). Monocytes therefore have multiple methods of recognizing S1, and the receptor(s) responsible for signaling to induce immunometabolic activation deserve further investigation.

The second major advance in this study is the identification of metformin as a potential immunometabolic regulator of inflammatory responses to SARS-CoV-2. Small molecule inhibitors of HIF-1α (chetomin) and glucose metabolism (2-deoxyglucose) blocked cytokine production in S1-treated monocytes, suggesting that interfering with downstream signaling pathways activated by spike protein binding is a potential therapeutic strategy to target inflammation during COVID-19. As these compounds are not approved for human use or have low efficacy in humans as described above, we evaluated the ability of metformin to suppress glycolytic reprogramming and cytokine production in S1-stimulated monocytes. Metformin reduced cytokine production and strongly inhibited both glycolysis and cellular respiration in culture, suggesting it as a potential treatment for hyperinflammation during COVID-19. Further, metformin blocked IL-6 production in monocytes infected with live SARS-CoV-2, suggesting this effect is not limited to artificial stimulation conditions with purified recombinant protein. However, we were unable to perform additional experiments using live SARS-CoV-2 to replicate further findings from this study, so a great deal of additional work is necessary to link our S1 data to our very preliminary SARS-CoV-2 observation.

Metformin is extremely inexpensive compared to many pharmaceuticals, with an estimated manufacturing cost under 10 USD per kg for the active ingredient (84) and a monthly wholesale cost as low as 25 USD (85). Metformin has been previously noted as a treatment for non-COVID acute respiratory distress syndrome (86) and is a potent suppressor of immune activation of monocytes and macrophages by other molecules including LPS (87–89). Additionally, several epidemiological studies have noted decreased mortality (90–94) and inflammation (systemic C-reactive protein) (95, 96) in COVID-19 patients who were taking metformin prior to diagnosis. These effects varied between studies, with mortality reductions of 20% - 80% across reports, but a meta-analysis found an overall reduction of 46% in mortality across 5 qualifying studies (94). Therefore, given these observations and its low cost, excellent safety profile, wide availability, and efficacy in inhibiting inflammatory responses to S1 *in vitro*, metformin is a promising candidate for further exploration as a COVID-19 therapeutic. Our study is limited to a single *in vitro* measure of metformin as a therapeutic for COVID-19, so a great deal of further study is necessary in order to establish this drug as a viable treatment. To this end, one recent study demonstrated metformin as efficacious in preventing acute respiratory distress syndrome in a preclinical animal model of COVID-19 (97). Therefore, although metformin is most likely to be useful as an adjuvant rather than front-line therapy for severe acute COVID-19, it remains an attractive option for targeting hyperinflammation in this disease to limit severity and mortality.

Limitations of this study include the small sample size, although the large effect sizes in most experiments are sufficient to convincingly demonstrate the interrogated mechanisms. More significantly, this study relies on healthy younger subjects and so does not address how comorbidities such as aging or obesity would affect the measured outcomes or the efficacy of the therapies we tested. We determined this to be appropriate, given our focus on mechanistic investigation in this study. However, further research is needed to determine if other populations have different responses to S1 and/or metformin. We additionally did not recruit with respect to race or sex and therefore have a relatively diverse sample which could increase variability. However, the magnitude of the responses did not show patterns consistent with race- or sex-based differences in our outcome measures. While it is conceivable that such differences exist, the effects are likely to be small enough that large studies would be necessary to detect small between-population differences. Finally, none of our subjects were previously taking metformin, and the ability of *in vivo* metformin to modulate monocyte responses to S1 requires further study.

## Conclusion

In summary, we demonstrate here that the spike protein subunit 1 from SARS-CoV-2 causes activation of HIF-1α dependent glycolysis and inflammatory cytokine production in monocytes which can be suppressed by treatment with the diabetes drug metformin. These experiments detail a mechanism by which SARS-CoV-2 mediates metabolic reprogramming previously described in human monocytes, and additionally provides a potential mechanism for the observation that metformin is protective against mortality in COVID-19 patients. Continued research in this area has the potential to define therapeutic strategies and additional molecular targets for the treatment of COVID-19-associated hyperinflammation.

## Data Availability Statement

The datasets presented in this study can be found in online repositories. The names of the repository/repositories and accession number(s) can be found below: https://figshare.com/, DOI: 10.6084/m9.figshare.c.5463525.

## Ethics Statement

The studies involving human participants were reviewed and approved by the University of Memphis Institutional Review Board. The patients/participants provided their written informed consent to participate in this study.

## Author Contributions

BP conceived the study. TC and BP designed experiments. TC, RE, JY, KD, and BP collected data. BP analyzed data and prepared the first manuscript draft. TC, RE, JY, KD, and BP edited the manuscript draft. All authors read and approved the final manuscript.

## Funding

The study was primarily supported by a University of Memphis/University of Tennessee Health Science Center Collaborative Research Network (CORNET) award to BP and TC, with additional support from American Heart Association grants 18AIREA33961089 and 19TPA34910232 to BP, and a University of Memphis College of Health Sciences faculty research grant to BP. RE was supported by a postdoctoral fellowship funded by the University of Memphis Division of Research and Innovation through the Carnegie R1 Postdoc Program.

## Conflict of Interest

The authors declare that the research was conducted in the absence of any commercial or financial relationships that could be construed as a potential conflict of interest.

## Publisher’s Note

All claims expressed in this article are solely those of the authors and do not necessarily represent those of their affiliated organizations, or those of the publisher, the editors and the reviewers. Any product that may be evaluated in this article, or claim that may be made by its manufacturer, is not guaranteed or endorsed by the publisher.
